# Development and validation of a machine learning model for preoperative prediction of intraoperative hypothermia in gynecological laparoscopic surgery: a two-center cohort study

**DOI:** 10.3389/fmed.2026.1839255

**Published:** 2026-09-28

**Authors:** Chunping Li, Bo Liu, Jianping Zhang, Fei Jia, Yong Tang

**Affiliations:** 1 Department of Anesthesiology, Sichuan Jinxin Xinan Women & Children’s Hospital, Chengdu, Sichuan, China; 2 Department of Anesthesiology, Chengdu Jinjiang District Women & Children Health Hospital, Chengdu, Sichuan, China

**Keywords:** gynecological laparoscopic, Intraoperative hypothermia, machine learning, Model interpretability, Preoperative prediction

## Abstract

**Background:**

Intraoperative hypothermia (IOH, temperature <36.0 °C) is a common complication in gynecological laparoscopic surgery that leads to significant adverse outcomes that would benefit from preoperative risk prediction.

**Methods:**

We developed and compared two predictive models—a conventional multivariable logistic regression (LR) model and a gradient boosting machine (XGBoost) model—to preoperatively predict IOH based on 20 objectively measurable preoperative clinical variables that could be directly extracted from electronic health records (EHRs). The derivation cohort consisted of 460 cases from a tertiary care center in 2024, which were randomly divided into a training set (*n* = 322) and a test set (*n* = 138) at a 7:3 ratio. For external validation, we used an independent cohort of 183 cases from a second medical institution during the same period. Model performance was evaluated using metrics including the area under the receiver operating characteristic curve (AUROC) and area under the precision-recall curve (AUPRC), with SHapley Additive exPlanations (SHAP) method applied for feature importance analysis.

**Results:**

We initially analyzed 20 preoperative variables. The final model incorporated eight key indicators: body mass index (BMI), ASA physical status, fasting period, basal body temperature, estimated surgical duration, hypertension status, serum albumin level, and alanine aminotransferase (ALT). The derivation cohort consisted of 460 patients, split into a training set (*n* = 322) and a test set (*n* = 138). An independent external validation cohort included 183 patients. By AUROC comparison, the XGBoost model outperformed the LR model in both the training set (0.933 vs. 0.891) and the external validation set (0.870 vs. 0.838), while performing slightly lower in the test set (0.821 vs. 0.865).

**Conclusions:**

We developed and validated an interpretable XGBoost model using eight readily available preoperative clinical indicators. The model demonstrated good performance for the preoperative prediction of IOH in gynecological laparoscopic surgery, which may facilitate early identification of high-risk patients and timely intervention.

## 1 Introduction

Intraoperative hypothermia (IOH), defined as a core temperature below 36.0 °C during surgery, is associated with adverse outcomes including bleeding, infection, cardiovascular complications, prolonged hospitalization, and increased costs (1–5). Although laparoscopic surgery is widely used in gynecology for its minimal invasiveness and rapid recovery, the incidence of IOH in these procedures remains high, reaching up to 50% (6).

Routine warming for low-risk patients may waste resources and increase adverse events (7). Accurately identifying high-risk patients is therefore crucial for effective resource allocation and targeted warming, improving efficiency and reducing surgical risks.

Preoperatively identifying high-risk patients is therefore essential for optimizing surgical safety and recovery (8). Ren et al. developed a conventional logistic regression nomogram for the same population (internal validation AUC: 0.810), but its reliance on an intraoperative variable (operative time) limits its utility for preoperative prediction (9). Machine learning has emerged as a promising tool for clinical prediction, yet existing models for IOH remain limited. Lu et al. relied primarily on intraoperative data (10), while Cao et al., though using preoperative variables, reported suboptimal accuracy (C-index: 0.712) in a heterogeneous surgical cohort (11).

Our study aims to develop and validate a machine learning model for the preoperative prediction of IOH in patients undergoing gynecological laparoscopic surgery, based on routinely available clinical indicators.

## 2 Methods

### 2.1 Study design

This multicentre retrospective study was approved by the Ethics Committee of Sichuan Jinxin Xinan Women & Children's Hospital [approval number: 2026(03)] and Chengdu Jinjiang District Women & Children Health Hospital (approval number: 202603). The requirement for obtaining written informed consent was waived due to the retrospective nature of the study. Additionally, this research adhered to the recommendations outlined in the “Guidelines for Development and Reporting Machine Learning Predictive Models in Biomedical Research: A Multidisciplinary View” (12). The reporting of this observational study follows the Strengthening the Reporting of Observational Studies in Epidemiology (STROBE) statement and the TRIPOD-AI (TRIPOD + AI) guidelines for prediction model studies using regression or machine learning methods.

### 2.2 Study population

In this study, we included adult female patients who underwent gynecological laparoscopic surgery. The exclusion criteria include having a preoperative temperature exceeding 37.5 °C or below 36.0 °C, a history of thyroid dysfunction (either hypothyroidism or hyperthyroidism), and patients with temperature regulation disorders, such as severe infections or central nervous system diseases. Notably, while patients with thyroid disorders were excluded, those with other endocrine diseases were not excluded from the analysis, as they were considered potential confounding variables and were included in the model development.

### 2.3 Data collection

All preoperative data were obtained from the EHR system. To construct the model, patient characteristics were collected, including age, BMI, American Society of Anesthesiologists (ASA) physical status, fasting period, and drinking prohibition period. Basal temperature and heart rate (HR) were also recorded. Surgical-related characteristics included the approach used (either single-port or multi-port laparoscopy) and whether the surgery was classified as an emergency. Additionally, we collected the estimated duration of surgery, which was documented by the attending surgeon during preoperative assessment based on the planned procedure complexity and prior surgical experience, and was recorded in the EHR prior to surgery, and the anesthesia induction time (categorized into three intervals: 8:00 to 15:59, 16:00 to 23:59, and 00:00 to 8:00).

In addition, the patients' previous surgical history and comorbidities (diabetes mellitus, hypertension, cardiovascular disease, respiratory disease, and endocrine disease) were extracted. Preoperative laboratory results collected included albumin, alanine aminotransferase (ALT), and aspartate aminotransferase (AST). Furthermore, the occurrence of intraoperative hypothermia (defined as a core temperature <36.0 °C) was recorded, as it is the primary outcome of the study.

### 2.4 Primary outcome

The primary outcome was the occurrence of IOH, defined as a core body temperature <36.0 °C at any point during laparoscopic gynecological surgery. Cases meeting this threshold were documented as outcome events. All patients underwent continuous wireless axillary temperature monitoring from operating room arrival until the end of the surgical procedure.

### 2.5 Model development

We developed two predictive models using the derivation cohort and validated it exclusively on an external cohort. Both models incorporate 20 readily extractable preoperative EHR variables—covering demographics, key surgical factors, comorbidities, and three laboratory parameters—all input in raw form without preprocessing.

We developed two predictive models: a conventional multivariable logistic regression (LR) model and a gradient boosting machine (XGBoost) model. The LR model served as a statistical baseline to assess the incremental value of the machine learning approach.

The XGBoost model was chosen for its robust binary classification, built-in handling of missing values, and regularization against overfitting (13). Missing values were handled natively by the gradient boosting algorithm. For each split node, the algorithm attempts to assign missing values to the left or right child node and selects the direction that minimizes the loss. This does not treat continuous variables as categorical, but allows missing values to follow the optimal direction learned from the data. Given the very low missing proportion (≤1.2%), multiple imputation was not applied.

The derivation cohort was randomly split into training and test sets (7:3) (14). In the training set, we applied the least absolute shrinkage and selection operator (LASSO) algorithm to select key predictors. LASSO identifies variables based on multivariable contribution and regularization, regardless of univariate significance (15). By sparsifying coefficients and reducing irrelevant features, it enhances interpretability and mitigates overfitting. The concise and clinically relevant feature set identified by LASSO was used for both models, thereby simplifying the model structure and potentially improving its external generalizability for real-world application. Predictor scaling or normalization was not performed prior to LASSO selection, as all continuous variables were measured on comparable scales and the subsequent gradient boosting algorithm is tree-based and invariant to feature scaling. Categorical variables were converted to dummy variables prior to LASSO regression.

All hyperparameter tuning procedures were performed exclusively on the training set using five-fold cross-validation. The test set and external validation set were not involved in any stage of model development or hyperparameter optimization. We determined the optimal hyperparameters for the gradient boosting algorithm through a comprehensive five-fold cross-validation coupled with randomized search, systematically evaluating multiple parameter combinations including learning rates (0.01, 0.03, 0.05, 0.07), class weights for positive samples (1, 10, 20, 30), maximum tree depths (3, 4, 5), number of estimators (50, 75, 100), subsampling ratios for instances (0.8, 1.0) and features (0.8, 1.0), along with gamma regularization values (0.5, 0.7, 0.9). The final hyperparameter configuration is provided in Supplementary Table S1.

### 2.6 Model validation

The performance of both models was evaluated and compared using various metrics, with the AUROC serving as the primary criterion for selecting the optimal model. Additionally, AUPRC, sensitivity, specificity, precision, balanced accuracy, and F1 score were also assessed. The optimal cut-off was determined using the Youden Index.

Decision curve analysis (DCA) was performed to assess the clinical utility and net benefit of the model. Calibration plots were used to compare predicted and observed risks of intraoperative hypothermia. Additionally, the SHAP method was applied to improve model interpretability, with SHAP values quantifying the contribution of each feature to predictions.

We developed an interactive web-based calculator (https://lcpapp.shinyapps.io/hypothermia-prediction) to provide real-time, individualized IOH risk assessment. The tool outputs a continuous probability score (0–100%). In practice, anesthesiologists can enter the preoperative variables for instant risk stratification, supporting timely decisions such as preoperative warming or intraoperative temperature monitoring.

### 2.7 Sample size

We used the “pmsampsize” R package (version 4.3.1; R Development Core Team, Vienna, Austria) to calculate the sample size. The calculation was based on c-statistics ranging from 0.712 to 0.850, 20 candidate parameters, and a prevalence of 50.0%, using the formula by Riley and colleagues (11, 16, 17). Considering a shrinkage of 0.9, the minimum sample size was determined to be 423–1,231. The sample size of our derivation cohort was 460, satisfying the above-mentioned criteria.

### 2.8 Statistical analysis

The Kolmogorov–Smirnov test was used to evaluate the normal distribution of continuous variables. For normally distributed continuous variables, data are presented as the mean ± standard deviation (SD) and were analyzed via the independent-sample t test. Nonnormally distributed continuous variables are expressed as the median (M) and interquartile range (IQR) and were compared using the Mann–Whitney *U*-test. Categorical variables are presented as numbers and percentages (%) and compared with the Chi-square test or Fisher's exact test as appropriate. A P-value of less than 0.05 was considered statistically significant.The model development and validation were performed using R version 4.3.1 and SPSS software version 25.0.

## 3 Results

### 3.1 Patients characteristics

From January 1, 2024 to June 30, 2024, a total of 643 patients were included in this study. Specifically, 498 patients were initially enrolled from Sichuan Jinxin Xinan Women & Children's Hospital, of whom 460 remained after applying the exclusion criteria to form the derivation cohort. This derivation cohort was then randomly divided at a 7:3 ratio into a training set (*n* = 322) for model development and a test set (*n* = 138) for internal validation. Separately, 201 patients were initially enrolled from Chengdu Jinjiang District Women & Children Health Hospital, with 183 ultimately constituting an independent external validation cohort (*n* = 183) for external validation (Figure 1).

**Figure 1:**
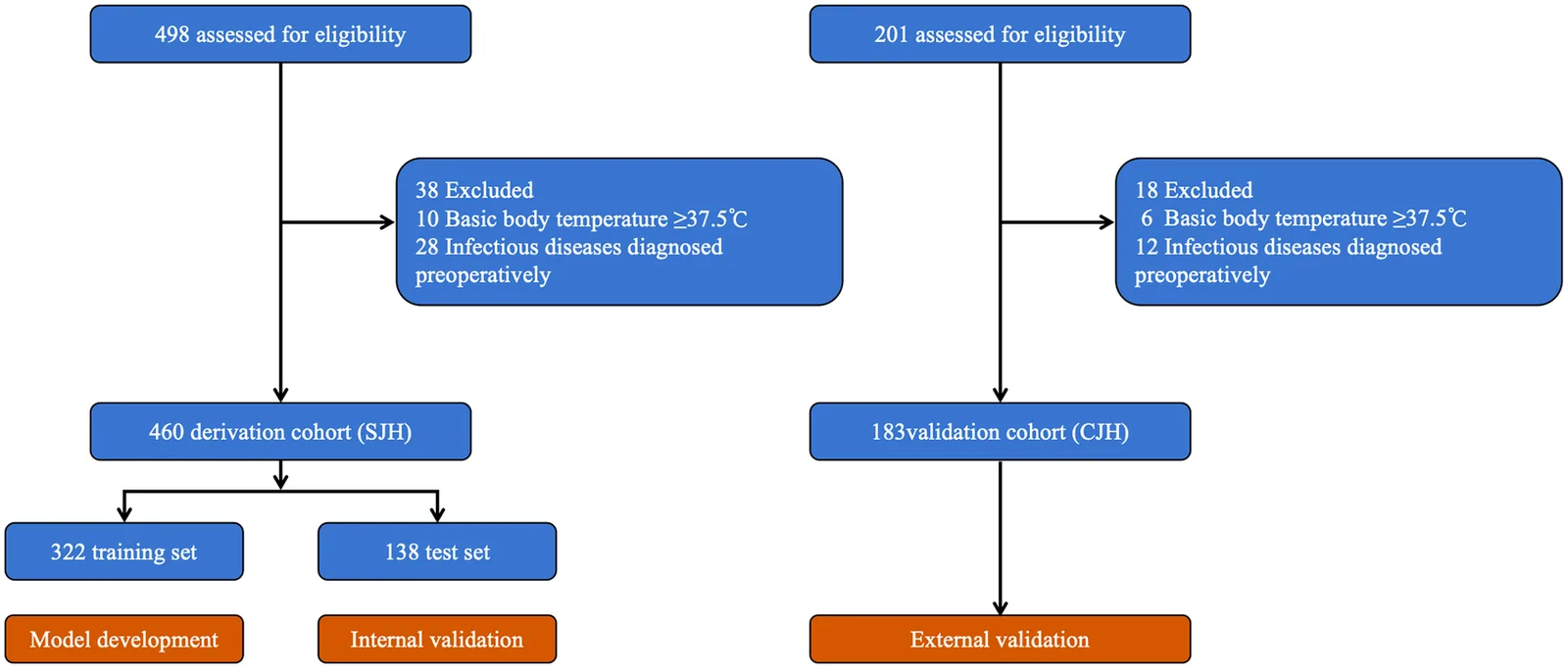
Study flowchart. SJH, Sichuan Jinxin Xinan Women & Children's Hospital; CJH, Chengdu Jinjiang District Women & Children Health Hospital.

The training set characteristics are shown in Table 1. Comparisons between training/test sets and between the derivation/external validation cohorts (with missing data rates) are provided in Supplementary Tables S2, S3, respectively.

**Table 1:** Comparison of characteristics in the training set of the derivation cohort.

Characteristics		IOH		*P* value
		Yes (131)	No (191)	
Patient characteristics				
Age (years)	0 (0)	36 (30–48)	33 (29–38)	<0.001
BMI (kg/m^2^)	0 (0)	21.8 (19.5–23.9)	21.6 (19.8–23.7)	0.963
ASA physical status (%)	0 (0)			<0.001
Ⅰ	0 (0)	80 (61.1)	149 (78.0)	
Ⅱ	0 (0)	46 (35.1)	42 (22.0)	
Ⅲ	0 (0)	5 (3.8)	0 (0.0)	
Fasting period (h)	4 (1.2)	12 (11–14)	12 (8–14)	0.097
Drinking prohibition period (h)	2 (0.6)	12 (11–14)	12 (8–14)	0.101
Basal temperature (℃)	4 (1.2)	36.4 (36.2–36.5)	36.6 (36.5–36.7)	<0.001
Basal HR (bpm)	0 (0)	77 (68–82)	79 (70 - 86)	<0.05
Previous surgical history (%)	0 (0)	66 (50.4)	80 (41.9)	0.140
Comorbidity				
Diabetes mellitus (%)	0 (0)	3 (2.3)	1 (0.5)	0.308
Hypertension (%)	0 (0)	12 (9.2)	3 (1.6)	<0.05
Cardiovascular disease (%)	0 (0)	2 (1.5)	1 (0.5)	0.569
Respiratory disease (%)	0 (0)	0 (0.0)	3 (1.6)	0.274
Endocrine disease (%)	0 (0)	4 (3.1)	5 (2.6)	1.000
Surgical-related				
Multi-port laparoscope (%)	0 (0)	82 (62.6)	120 (62.8)	0.966
Emergency (%)	0 (0)	26 (19.8)	71 (37.2)	<0.001
Estimated duration of surgery (h)	0 (0)	2 (1.5 - 3)	1 (1.0 - 1.5)	<0.001
Anesthesia induction time (%)				<0.05
8:00 to 15:59	0 (0)	114 (87.0)	149 (78.0)	
16:00 to 23:59	0 (0)	13 (9.9)	40 (20.9)	
00:00 to 8:00	0 (0)	4 (3.1)	2 (1.0)	
Laboratory tests				
Albumin (g/L)	0 (0)	43.4 (41.3– 45.7)	43.9 (42.5–45.7)	0.066
ALT (U/L)	0 (0)	12 (9–19)	12 (8–17)	0.117
AST (U/L)	0 (0)	20 (17–24)	19 (17–23)	<0.05

IOH, intraoperative hypothermia; BMI, body mass index; ASA, American society of anesthesiologists; HR, heart rate; ALT, alanine aminotransferase; AST, aspartate aminotransferase.

### 3.2 Primary outcome and predictive factor

The incidence of IOH was 40.7% (131/322) in the training set, 29.0% (40/138) in the test set, and 39.3% (72/183) in the validation cohort. LASSO regression uses a penalty function controlled by the hyperparameter *λ* to shrink coefficients and select key variables. Through ten-fold cross-validation, we identified the optimal *λ* (lambda min), which minimizes the mean squared error (MSE) (Figure 2A). Figure 2B shows the coefficient paths of 20 IOH-related features, demonstrating how stronger penalties enhance variable selection. Ultimately, eight key predictors were selected: BMI, ASA physical status, fasting period, basal temperature, estimated surgical duration, hypertension, albumin, and ALT.

**Figure 2:**
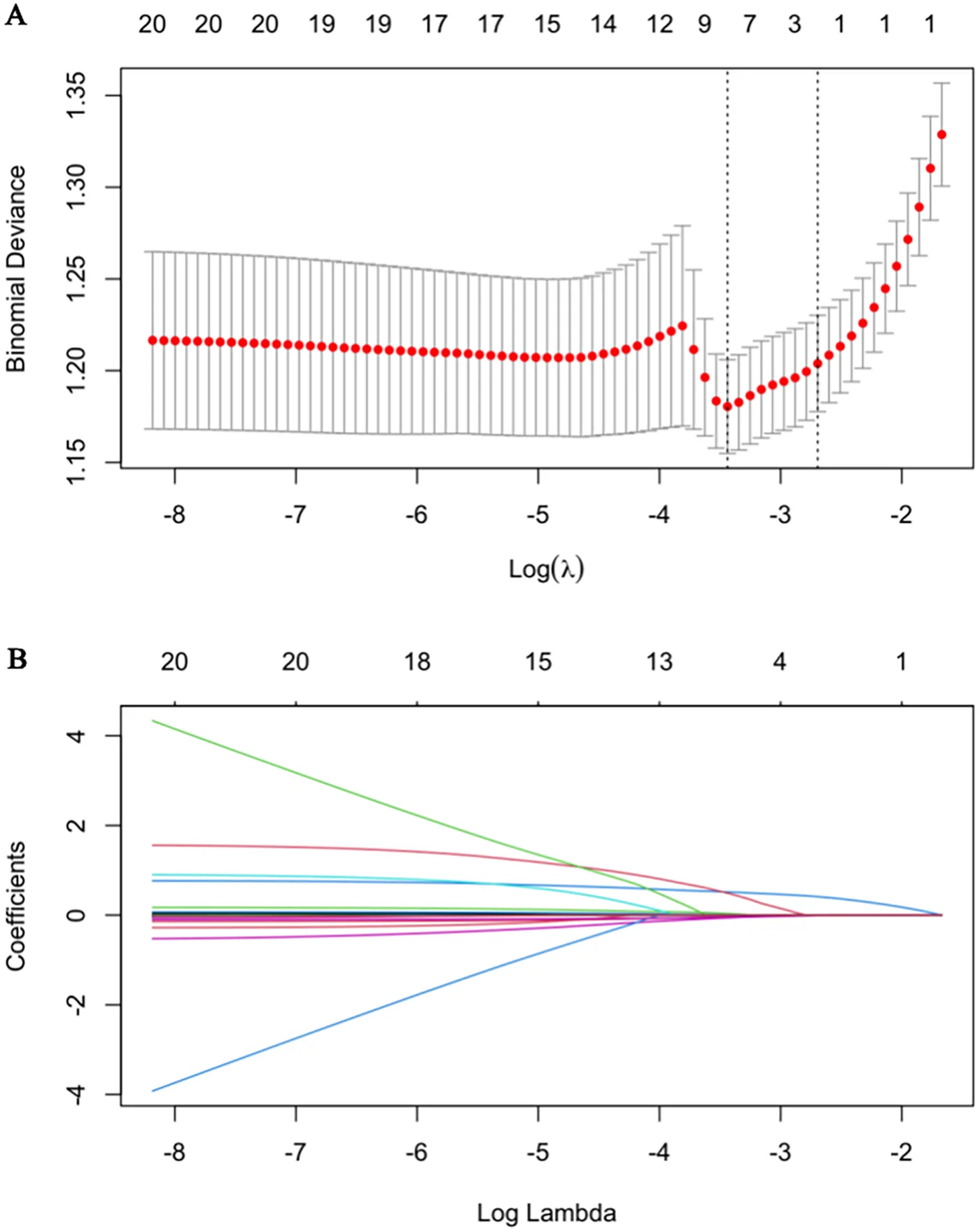
Lasso regression analysis. **(A)** The graph displays the cross-validated MSE as it relates to the logarithm of the regularization parameter (*λ*). The red dashed line marks the selected *λ* value, referred to as “lambda min”, which corresponds to the point of minimal MSE. The gray bars indicate the standard error associated with each *λ* value. **(B)** The coefficient paths for 20 features associated with IOH are shown as *λ* increases. Each line corresponds to the coefficients of an individual feature, illustrating how significant variables are maintained while less important ones are reduced towards zero.

### 3.3 Machine learning prediction of IOH

By AUROC comparison, the XGBoost model outperformed the conventional LR model in both the training set (0.933 vs. 0.891) and the external validation set (0.870 vs. 0.838), while performing slightly lower than LR in the test set (0.821 vs. 0.865) (Figure 3). In the training and external validation sets, the XGBoost model also demonstrated superior performance over the LR model across other evaluation metrics, including AUPRC, sensitivity, specificity, precision, balanced accuracy, and F1 score (Table 2). Therefore, based on comprehensive consideration of discriminative ability, classification balance, and clinical applicability, the XGBoost model was selected as the final predictive model. In addition to the full model, we evaluated a simplified model excluding estimated surgical duration. This model achieved an AUROC of 0.864 (95% CI: 0.811–0.916) in the external validation set (Supplementary Table S4), indicating that the remaining seven predictors still provide robust prediction when estimated duration is unavailable.

**Figure 3:**
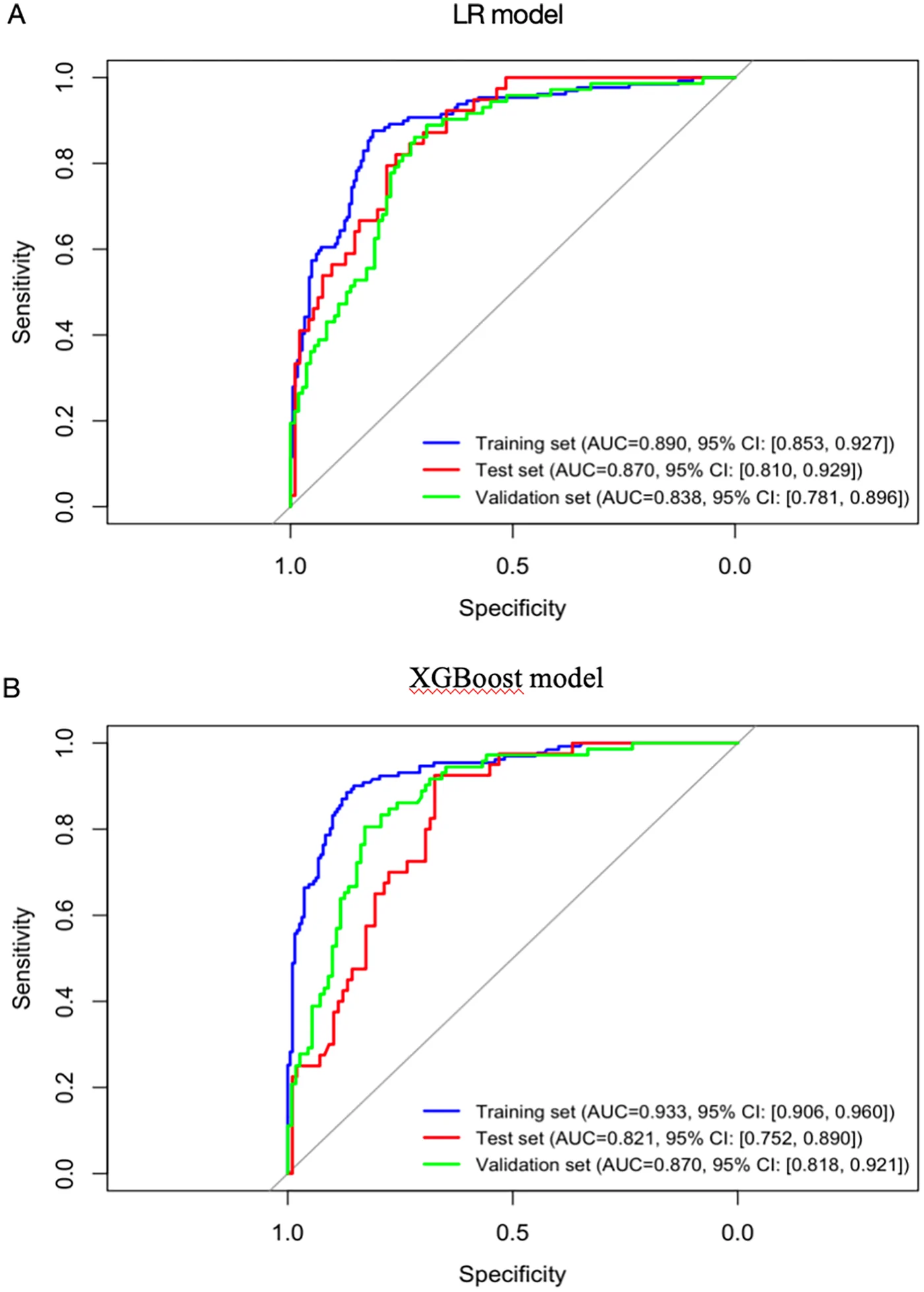
Receiver operating characteristic (ROC) curves for the LR model **(A)** and XGBoost model **(B)** in the training, test, and validation sets, demonstrating the comparative discriminative performance of the two models.

**Table 2:** Model evaluation results on different sets.

Model	Characteristics	AUROC	AUPRC	Sensitivity	Specificity	Precision	Balanced accuracy	F1 score
LR model	Training set	0.891 (0.854–0.927)	0.852 (0.820–0.882)	0.790 (0.740–0.835)	0.833 (0.790–0.872)	0.748 (0.695–0.795)	0.812 (0.775–0.846)	0.769 (0.725–0.810)
	Test set	0.865 (0.804–0.925)	0.716 (0.665–0.765)	0.587 (0.500–0.675	0.859 (0.800–0.918)	0.675 (0.590–0.755)	0.723 (0.670–0.775)	0.628 (0.555–0.700)
	Validation set	0.838 (0.780–0.895)	0.768 (0.720–0.812)	0.690 (0.620–0.760)	0.795 (0.730–0.855)	0.681 (0.610–0.750)	0.742 (0.690–0.795)	0.685 (0.620–0.745)
XGBoost model	Training set	0.933 (0.906–0.960)	0.911 (0.884–0.938)	0.823 (0.758–0.888)	0.917 (0.878–0.956)	0.885 (0.838–0.932)	0.870 (0.833–0.907)	0.853 (0.808–0.898)
	Test set	0.821 (0.752–0.890)	0.592 (0.521–0.663)	0.509 (0.426–0.592)	0.864 (0.800–0.928)	0.725 (0.645–0.805)	0.686 (0.628–0.744)	0.598 (0.523–0.673）
	Validation set	0.870 (0.818–0.921)	0.791 (0.736–0.846)	0.753 (0.686–0.820)	0.868 (0.808–0.928)	0.806 (0.744–0.868)	0.811 (0.761–0.861)	0.778 (0.718–0.838)

AUROC, area under the receiver operating characteristic curve; AUPRC, area under the precision-recall curve; LR, logistic regression; XGBoost, gradient boosting machine.

### 3.4 Model calibration and clinical utility

Calibration curves for the XGBoost model are shown in Supplementary Figure S1, and decision curve analysis results are shown in Figure 4. Calibration curves indicated varying agreement between predicted and observed risks: the training set showed some deviation, while the test and validation sets aligned more closely with the ideal line (Supplementary Figure S1). In the training set, the calibration intercept was −0.002, calibration slope was 0.871, and calibration-in-the-large was −0.002. In the test set, the calibration intercept was 0.004, calibration slope was 0.762, and calibration-in-the-large was 0.004. In the validation set, the calibration intercept was −0.007, calibration slope was 0.913, and calibration-in-the-large was −0.007. Decision curve analysis revealed significant net clinical benefit across a broad threshold range (approximately 0–60%) for the training and validation sets, with a somewhat narrower benefit range for the test set. Additionally, we performed the Hosmer–Lemeshow test in the validation set, which yielded a chi-squared value of 13.175 (df = 8, *p* = 0.106).

**Figure 4:**
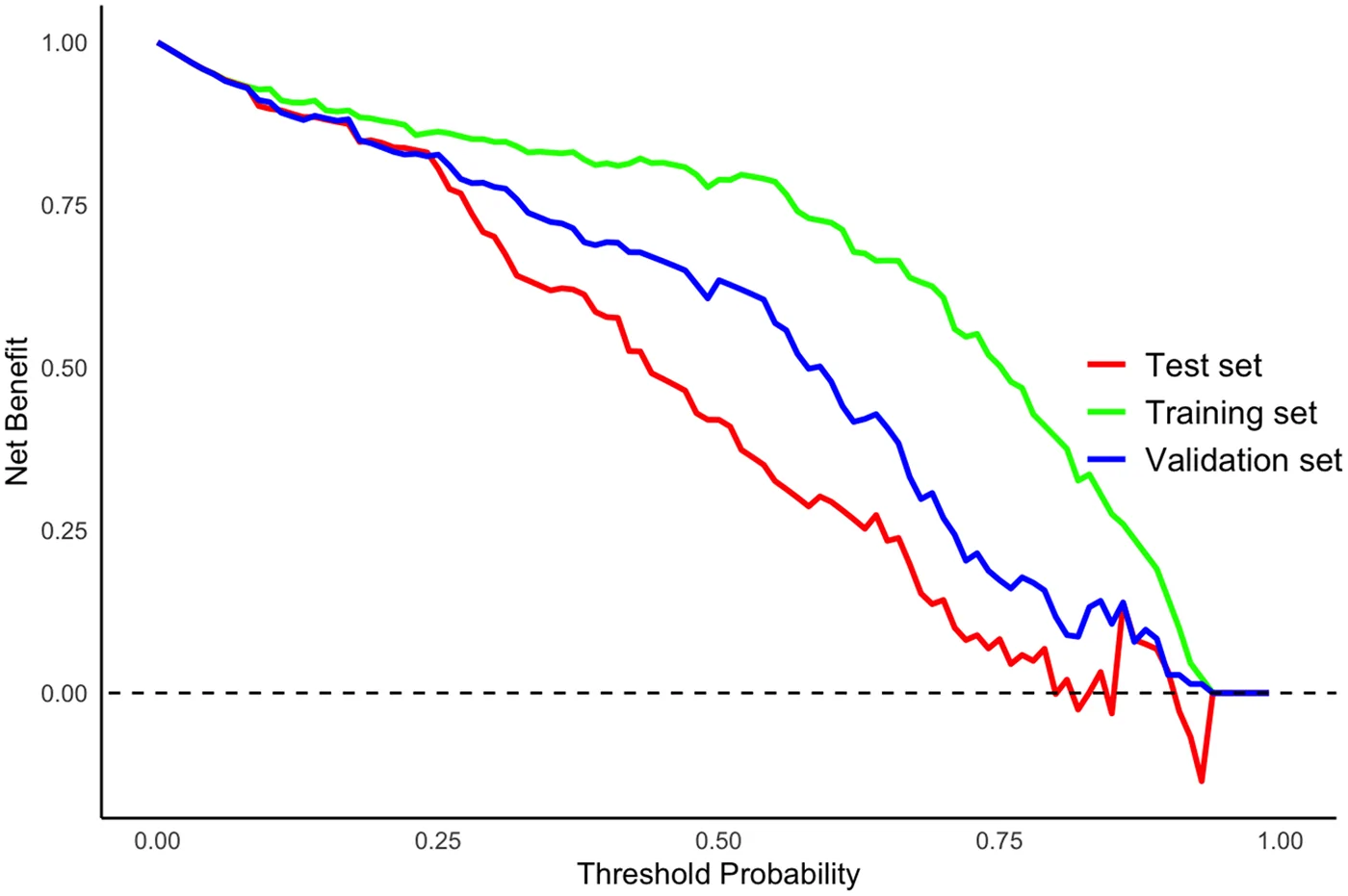
Decision curve analysis: the decision curve analysis (DCA) illustrates the net benefit of using the predictive model across different threshold probabilities for various datasets. The curves represent the net benefit for the training set (green), testing set (red), and validation set (blue). The dotted horizontal line at a net benefit of 0 indicates the scenario where no action is taken. The area above this line reflects the value of utilizing the model for decision-making. The divergence of the curves from this baseline demonstrates the model's effectiveness in enhancing net benefit at varying threshold levels, thereby assisting in identifying optimal thresholds for clinical application across the sets.

### 3.5 Shap analysis

The SHAP summary plot (Figure 5) demonstrates the contribution of eight preoperative features to the predicted risk of IOH during gynecological laparoscopy. Estimated operative time and basal body temperature were the two most influential predictors, followed by serum albumin, BMI, preoperative fasting duration, ALT, ASA physical status, and hypertension history. SHAP dependency plots depicting the relationship between each feature and the outcome are presented in Supplementary Figure S2.

**Figure 5:**
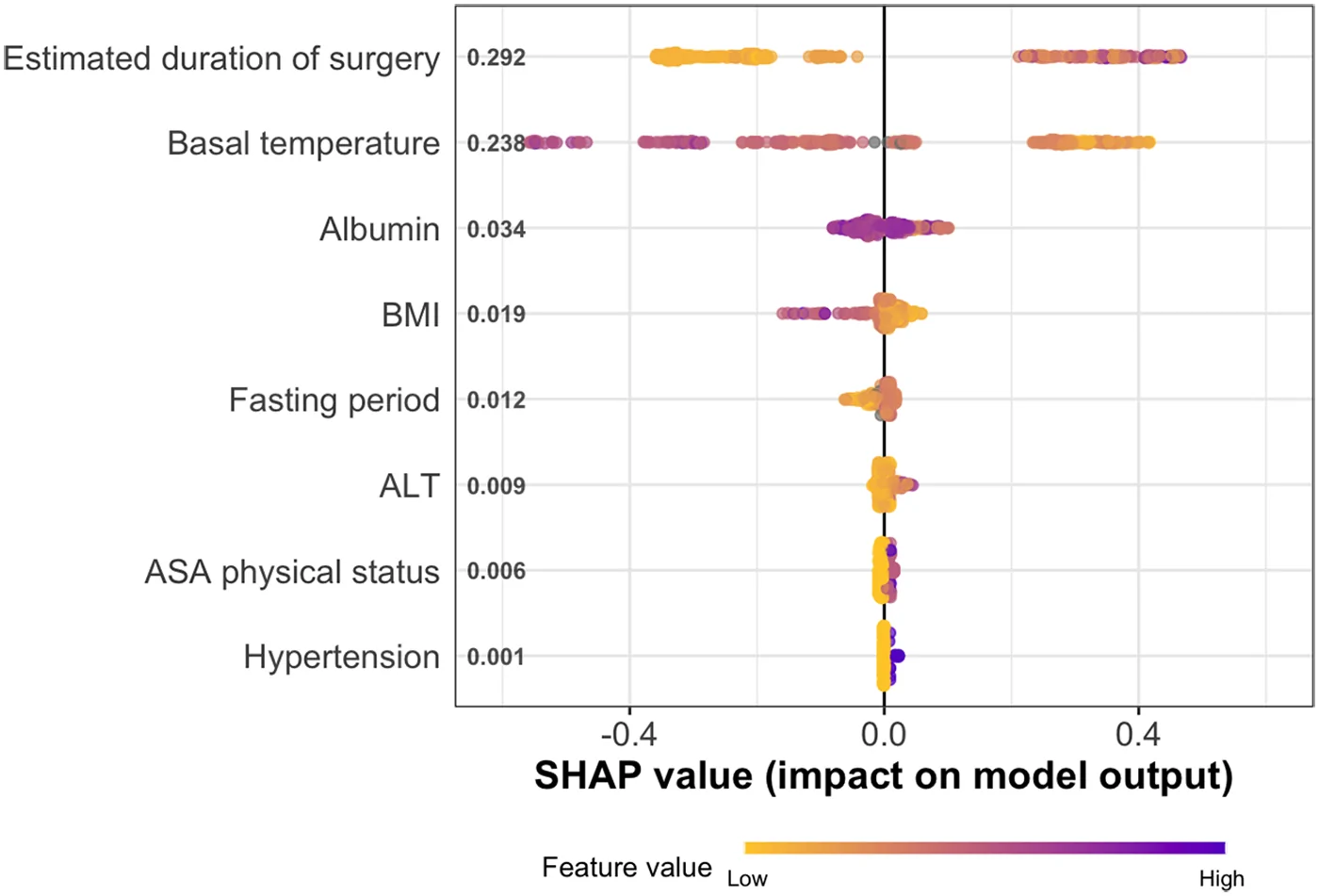
Shap summary plot illustrating the eight predictive features in the final model. Each point represents a patient. The position on the *x*-axis shows the feature's impact on the model output (SHAP value), with values further from zero indicating a stronger influence. Point color indicates the feature's original value: purple for higher values, yellow for lower values. Features are ranked from top to bottom by their mean absolute SHAP value (global importance).

### 3.6 Sensitivity analyses

In the sensitivity analysis, excluding the estimated duration of surgery reduced the AUROC of the external validation set to 0.864 (95% CI: 0.811–0.916), while excluding basal temperature resulted in an AUROC of 0.866 (95% CI: 0.812–0.919). The performance declines observed after excluding these two dominant predictors were more pronounced than for any other features. In contrast, the exclusion of any other single preoperative variable led to only marginal reductions in AUROC across all datasets (Supplementary Table S4).

## 4 Discussion

We developed and externally validated an XGBoost model to preoperatively predict IOH in gynecological laparoscopic surgery. Using eight routinely available preoperative EHR indicators, the model achieved good discrimination. SHAP analysis provided clear feature contribution visualization. Model parameters and a web-based calculator are provided for clinical implementation.

A key strength of our model is its reliance on only eight readily available preoperative clinical indicators extracted from EHRs, eliminating the need for subjective clinician assessment. More importantly, it operates entirely preoperatively, enabling early intervention by avoiding the delay inherent in models that rely on intraoperative data. The model automates IOH risk stratification, reducing bias and inconsistencies (18). For clinical use, we set a threshold (e.g., 50%) in our web-based calculator to identify high-risk patients for initiating preventive warming measures.

Many machine learning models in clinical practice are hindered by excessive input variables, which reduce generalizability when data is incomplete (19, 20). In contrast, our model intentionally minimized complexity through LASSO regression, selecting only eight key preoperative predictors. This streamlined design ensures adaptability across diverse clinical settings without compromising accuracy. The model's simplicity, reliance on objective EHR data, and ability to provide timely preoperative risk assessment make it a practical tool for widespread use in gynecological laparoscopy.

External validation is critical for evaluating model reproducibility and generalizability (21). Nevertheless, performance often declines due to heterogeneity in patient populations, clinical practices, and data collection (22). In our study, the model achieved good accuracy in the derivation cohort but declined in the test set. This drop may stem from differences in surgical protocols, anesthesia management, patient characteristics, and inconsistencies in data collection. The most notable decline occurred in the test set, potentially due to greater patient heterogeneity, inclusion of different surgery types, varying distributions of preoperative indicators, and the set's relatively small sample size, which can affect statistical stability. Sensitivity analyses confirmed that key predictors significantly influence model performance.

SHAP analysis is crucial for explaining how input variables affect model predictions (23). In our study, the SHAP plot identified estimated surgery duration and basal temperature as the top predictors of IOH. These visualizations link complex model outputs to actionable clinical insights, helping anesthesiologists understand and trust the model's decisions.

Although gradient boosting can handle missing values, their presence may still affect prediction (24). The exclusion of key variables notably reduces performance, underscoring their importance. To ensure accuracy across hospitals, we recommend strict data collection protocols to minimize missing preoperative indicators, especially for the eight key predictors. Future EHR integrations could also include real-time alerts for incomplete data.

Most model inputs align with known IOH risk factors (10, 25–27). Estimated surgery duration was the strongest predictor, consistent with evidence linking longer operations to higher hypothermia risk. Prolonged anesthesia disrupts thermoregulation, typically lowering core temperature by 1–2 °C, and extended exposure to cool operating rooms worsens heat loss (2, 28). We acknowledge that this variable is a surgeon-estimated procedural factor rather than an objective patient characteristic, which may introduce variability. However, this estimate is routinely documented for surgical scheduling and, combined with seven other preoperative variables, our model achieved robust external validation (AUROC 0.870). Although subjective, the “estimated” (rather than “actual”) duration is clinically crucial, as it enables preoperative risk assessment and timely intervention—the core aim of our model. Nevertheless, the simplified model excluding this variable showed only a slight performance decrease (external validation AUROC: 0.870 to 0.864), supporting the clinical utility of the simplified model when estimated duration is unavailable. Our web-based calculator also accommodates this by allowing users to omit this input while still obtaining a reliable risk prediction.

Although multiple preoperative variables were initially considered, only estimated surgery duration was retained as a surgery-specific risk factor, with sensitivity analysis confirming its critical role. Future research should refine predictions by subclassifying surgeries and incorporating more procedure-related details.

Our model incorporated ALT but did not include AST. Compared with AST (which is also distributed in cardiac and skeletal muscle), ALT is more liver-specific, and its elevation often reflects subclinical hepatic steatosis or mild liver dysfunction (29, 30). Such liver-associated alterations may indirectly disrupt intraoperative thermal balance by affecting systemic metabolism, inflammatory status, or autonomic regulation.

Calibration is essential for validating clinical prediction models, as it evaluates the agreement between predicted and observed outcomes (31). Our model demonstrated good calibration in the derivation cohort, though external validation revealed slight deviations, particularly for high-risk predictions. These differences likely reflect inherent variations in patient populations and institutional practices between medical centers.

Decision curve analysis demonstrated that the model provided a positive net benefit across a wide range of threshold probabilities (approximately 0–60%) in the training and external validation cohorts, supporting its clinical utility for guiding preventive warming interventions.

Our model serves as a valuable preoperative screening tool to identify high-risk IOH patients and guide targeted warming interventions. While precision may vary across settings—resulting in some false positives—its ability to flag high-risk cases remains crucial for reducing IOH-related complications. Given the low-cost, noninvasive nature of preventive warming, intervening in high-risk patients can meaningfully improve surgical safety. Moreover, the model's interpretability (via SHAP analysis) offers clinicians and patients clear insights into risk contributors, supporting informed, collaborative decisions on personalized care.

Several factors may explain why the model performed better in the external validation cohort (AUROC: 0.870) than in the internal test set (AUROC: 0.821). First, the test set had a substantially lower IOH incidence (29.0% vs. 39.3% in the external cohort), which likely contributed to its lower AUPRC, as this metric is sensitive to prevalence. Second, significant between-center differences in patient characteristics were observed (Supplementary Table S3), which may influence performance. Third, the test set comprised only 138 cases from a single split and may be subject to greater sampling variability than the external cohort. Importantly, the strong external performance (AUROC: 0.870; AUPRC: 0.791) suggests that the model captures generalizable patterns rather than overfitting to the derivation cohort, and the similar IOH prevalence between cohorts argues against the external set being intrinsically easier to classify.

This study has limitations. First, its retrospective design may introduce bias, necessitating future prospective multicenter validation. Second, we excluded patients with abnormal preoperative temperatures or thyroid dysfunction, restricting the model's applicability to those groups. Third, although the study was conducted at two hospitals, both are located within the same city, which limits geographic and demographic diversity and may restrict broader generalizability. Fourth, the estimated surgical duration is a subjective variable, which may introduce variability depending on the surgeon's estimation practice. Fifth, although the overall missing data proportion was very low (≤1.2%), missing values were treated as a separate category in gradient boosting, which may introduce bias if missingness is not completely at random. Sixth, we defined IOH as a core temperature <36.0 °C at any point during surgery, but this definition does not distinguish between transient redistribution hypothermia and sustained hypothermia. Seventh, the split-sample validation approach may result in greater variability in performance estimates compared with repeated cross-validation or bootstrap methods. Although we employed five-fold cross-validation for hyperparameter tuning and included an independent external validation cohort to assess generalizability, we acknowledge that repeated cross-validation or bootstrap-based internal validation with optimism correction would provide more robust performance estimates. These methods should be considered in future studies to further validate and refine the model. In addition, optimism correction was not performed, and although LASSO selection was performed only once for simplicity, we acknowledge this may underestimate selection variability. Future studies should embed the complete pipeline within each resampling iteration to better assess feature-selection stability and obtain corrected estimates. Finally, unmeasured factors (e.g., specific surgery type, operating room temperature, active warming protocols) could affect results. Perioperative warming protocols were not standardized or recorded in detail in this study, and potential differences between hospitals may influence the model's transportability across institutions. Future prospective, multicenter studies with larger samples—including excluded populations and more covariates—are needed to systematically evaluate the model's utility across diverse settings. In particular, external validation in healthcare systems from different geographic regions and with varied patient populations is required to confirm its broader generalizability.

## 5 Conclusion

In summary, we developed and externally validated an interpretable XGBoost model for the preoperative prediction IOH in gynecological laparoscopic surgery, using eight routinely available preoperative clinical indicators. The model demonstrated good discriminative performance across both internal and external validation cohorts. This preoperative framework may assist in early risk stratification and support timely interventions before surgery, though further validation in larger, more diverse cohorts is needed.

## Data Availability

The raw data supporting the conclusions of this article will be made available by the authors, without undue reservation.
