# Supplementary material for: Development and validation of a machine learning model for preoperative prediction of intraoperative hypothermia in gynecological laparoscopic surgery: a two-center cohort study
Source: Front Med (Lausanne). 2026 Sep 28;13:1839255. doi: 10.3389/fmed.2026.1839255 (PMC13654964; doi:10.3389/fmed.2026.1839255)
Supplement: Supplementary file 1 [file Supplementaryfile1.docx]

a	b

c	d

e	f

g	h

****Figure S2**. SHAP dependence plots for the eight features of the final model.** (a) BMI, (b) ASA physical status, (c) Fasting period, (d) Basal body temperature, (e) Estimated duration of surgery, (f) Hypertension status, (g) Serum albumin, (h) ALT. In the SHAP dependence plot, the impact of a feature on the model's prediction for IOH risk is visualized. Each dot represents a single patient prediction. The horizontal axis shows the actual value of the feature, while the vertical axis represents the corresponding SHAP value. The color gradient in this figure represents the magnitude of the feature values, ranging from low values (dark blue) to high values (yellow).
