# Supplementary material for: Development and validation of a machine learning model for preoperative prediction of intraoperative hypothermia in gynecological laparoscopic surgery: a two-center cohort study
Source: Front Med (Lausanne). 2026 Sep 28;13:1839255. doi: 10.3389/fmed.2026.1839255 (PMC13654964; doi:10.3389/fmed.2026.1839255)
Supplement: Supplementary file 2 [file Supplementaryfile2.docx]

**Figure S1.** Calibration Curve: The calibration curve illustrates the relationship between the predicted probabilities of the model and the actual observed outcomes across different sets. The dashed line indicates the ideal calibration scenario, where the predicted probabilities closely align with the observed proportions. The degree of deviation from this line reflects the accuracy and calibration effectiveness of the model across the various sets.
