# Supplementary material for: Development and validation of a machine learning model for preoperative prediction of intraoperative hypothermia in gynecological laparoscopic surgery: a two-center cohort study
Source: Front Med (Lausanne). 2026 Sep 28;13:1839255. doi: 10.3389/fmed.2026.1839255 (PMC13654964; doi:10.3389/fmed.2026.1839255)
Supplement: Supplementary file 3 [file Table1.docx]

**Table S1. Final Hyperparameter Configuration of the Gradient Boosting Model**

| Hyperparameter | Value |
| --- | --- |
| Learning rate | 0.05 |
| Number of estimators | 75 |
| Maximum tree depth | 4 |
| Class weight (positive) | 10 |
| Subsample ratio (instances) | 0.8 |
| Subsample ratio (features) | 0.8 |
| Gamma 0.5 | 0.5 |
