# Supplementary material for: Development and validation of a machine learning model for preoperative prediction of intraoperative hypothermia in gynecological laparoscopic surgery: a two-center cohort study
Source: Front Med (Lausanne). 2026 Sep 28;13:1839255. doi: 10.3389/fmed.2026.1839255 (PMC13654964; doi:10.3389/fmed.2026.1839255)
Supplement: Supplementary file 4 [file Table2.docx]

Compared to the training set, patients in the test set were older, with generally shorter fasting and drinking prohibition periods (P < 0.05), while no significant differences were observed in all other characteristics compared (P > 0.05) (Table S1).

**Tables S2. Characteristics Comparison between Training set and Test set**

| **Characteristics** | **Missing rate (%)** | **Training set**  **(n = 322)** | **Test set**  **(n = 138)** | ***P* value** |
| --- | --- | --- | --- | --- |
| **Patient characteristics** |  |  |  |  |
| Age (years) | 0 (0) | 34 (29 - 41) | 36 (31 - 43) | 0.026 |
| BMI (kg/m^2^) | 0 (0) | 21.6 (19.6 -23.8) | 22.0 (19.5 -24.3) | 0.441 |
| ASA physical status (%) |  |  |  | 0.241 |
| Ⅰ | 0 (0) | 229 (71.1) | 88 (63.8) |  |
| Ⅱ | 0 (0) | 88 (27.3) | 46 (33.3) |  |
| Ⅲ | 0 (0) | 5 (1.6) | 4 (2.9) |  |
| Fasting period (h) | 5 (1.0) | 12 (10 - 14) | 12 (8- 13) | ＜0.05 |
| Drinking prohibition period (h) | 4 (0.8) | 12 (9 - 14) | 12 (8- 13) | ＜0.05 |
| Basal temperature (℃) | 6 (1.3) | 36.5 (36.4 -36.6) | 36.5 (36.4 - 36.6) | 0.395 |
| Basal HR (bpm) | 0 (0) | 79 (70-84) | 80 (72-87) | 0.110 |
| Previous surgical history (%) | 0 (0) | 146 (45.3) | 64 (46.4) | 0.838 |
| **Comorbidity** |  |  |  |  |
| Diabetes mellitus (%) | 0 (0) | 4 (1.2) | 3 (2.2) | 0.433 |
| Hypertension (%) | 0 (0) | 15 (4.7) | 9 (6.5) | 0.492 |
| Cardiovascular disease (%) | 0 (0) | 3 (0.9) | 2 (1.4) | 0.639 |
| Respiratory disease (%) | 0 (0) | 3 (0.9) | 1 (0.7) | 1.000 |
| Endocrine disease (%) | 0 (0) | 9 (2.8) | 9 (6.5) | 0.069 |
| **Surgical-related** |  |  |  |  |
| Multi-port laparoscope (%) | 0 (0) | 202 (62.7) | 97 (70.3) | 0.136 |
| Emergency (%) | 0 (0) | 97 (30.1) | 43 (31.2) | 0.826 |
| Estimated duration of surgery (h) | 0 (0) | 1.5 (1-2.5) | 1.5 (1-2.6) | 0.650 |
| Anesthesia induction time(%) |  |  |  | 0.595 |
| 8:00 to 15:59 | 0 (0) | 263 (81.7) | 108 (78.3) |  |
| 16:00 to 23:59 | 0 (0) | 53 (16.5) | 28 (20.3) |  |
| 00:00 to 8:00 | 0 (0) | 6 (1.9) | 2 (1.4) |  |
| **Laboratory tests** |  |  |  |  |
| Albumin (g/L) | 0 (0) | 43.7 (42.0-45.7) | 43.9 (42.2-45.8) | 0.712 |
| ALT (U/L) | 0 (0) | 12 (8-17) | 12 (8-19) | 0.876 |
| AST(U/L) | 0 (0) | 19 (17-23) | 19 (16-23) | 0.997 |

Abbreviations: IOH=Intraoperative hypothermia, BMI=Body mass index, ASA=American society of anesthesiologists, HR=Heart rate, ALT=Alanine aminotransferase, AST=Aspartate aminotransferase.
