# Supplementary material for: Development and validation of a machine learning model for preoperative prediction of intraoperative hypothermia in gynecological laparoscopic surgery: a two-center cohort study
Source: Front Med (Lausanne). 2026 Sep 28;13:1839255. doi: 10.3389/fmed.2026.1839255 (PMC13654964; doi:10.3389/fmed.2026.1839255)
Supplement: Supplementary file 5 [file Table3.docx]

Compared to the derivation cohort, patients in the validation cohort had a higher level of albumin (P < 0.05) , while no significant differences were observed in all other characteristics compared (P＞0.05) (Table S2).

**Tables S3. Characteristics Comparison between Derivation and Validation Cohorts.**

| **Characteristics** | **Missing rate (%)** | **Derivation Cohort**  **(n = 460)** | **Validation Cohort**  **(n = 183)** | ***P* value** |
| --- | --- | --- | --- | --- |
| **Patient characteristics** |  |  |  |  |
| Age (years) | 0 (0) | 35 (29 - 41) | 35 (29 - 41) | 0.847 |
| BMI (kg/m^2^) | 0 (0) | 21.6 (19.6 -23.9) | 21.5 (19.5 -23.2) | 0.352 |
| ASA physical status (%) |  |  |  | 0.718 |
| Ⅰ | 0 (0) | 317 (68.9) | 129 (70.5) |  |
| Ⅱ | 0 (0) | 134 (29.1) | 49 (26.8) |  |
| Ⅲ | 0 (0) | 9 (2.0) | 5 (2.7) |  |
| Fasting period (h) | 5 (0.7) | 12 (9 - 14) | 12 (10- 14) | 0.233 |
| Drinking prohibition period (h) | 4 (0.6) | 12 (9 - 13) | 12 (10- 13) | 0.392 |
| Basal temperature (℃) | 6 (0.9) | 36.5 (36.4 -36.6) | 36.5 (36.4 - 36.6) | 0.436 |
| Basal HR (bpm) | 0 (0) | 79 (70-85) | 79 (70-84) | 0.522 |
| Previous surgical history (%) | 0 (0) | 210 (45.7) | 68 (37.2) | 0.053 |
| **Comorbidity** |  |  |  |  |
| Diabetes mellitus (%) | 0 (0) | 7 (1.5) | 5 (2.7) | 0.337 |
| Hypertension (%) | 0 (0) | 24 (5.2) | 14 (7.7) | 0.266 |
| Cardiovascular disease (%) | 0 (0) | 5 (1.1) | 3 (1.6) | 0.694 |
| Respiratory disease (%) | 0 (0) | 4 (0.9) | 2 (1.1) | 0.680 |
| Endocrine disease (%) | 0 (0) | 18 (3.9) | 12 (6.6) | 0.152 |
| **Surgical-related** |  |  |  |  |
| Multi-port laparoscope (%) | 0 (0) | 299 (65.0) | 114 (62.3) | 0.525 |
| Emergency (%) | 0 (0) | 140 (30.4) | 44 (24.0) | 0.122 |
| Estimated duration of surgery (h) | 0 (0) | 1.5 (1-2.5) | 1.5 (1-2.5) | 0.812 |
| Anesthesia induction time(%) |  |  |  | 0.123 |
| 8:00 to 15:59 | 0 (0) | 371 (80.7) | 160 (87.4) |  |
| 16:00 to 23:59 | 0 (0) | 81 (17.6) | 21 (11.5) |  |
| 00:00 to 8:00 | 0 (0) | 8 (1.7) | 2 (1.1) |  |
| **Laboratory tests** |  |  |  |  |
| Albumin (g/L) | 0 (0) | 43.8 (42.1-45.7) | 44.3 (42.5-46.5) | 0.022 |
| ALT (U/L) | 0 (0) | 12 (8-18) | 12 (9-19) | 0.318 |
| AST(U/L) | 0 (0) | 19 (17-23) | 20 (17-24) | 0.318 |

Abbreviations: IOH=Intraoperative hypothermia, BMI=Body mass index, ASA=American society of anesthesiologists, HR=Heart rate, ALT=Alanine aminotransferase, AST=Aspartate aminotransferase.
