# Supplementary material for: Development and validation of a machine learning model for preoperative prediction of intraoperative hypothermia in gynecological laparoscopic surgery: a two-center cohort study
Source: Front Med (Lausanne). 2026 Sep 28;13:1839255. doi: 10.3389/fmed.2026.1839255 (PMC13654964; doi:10.3389/fmed.2026.1839255)
Supplement: Supplementary file 6 [file Table4.docx]

The results of the sensitivity analysis are summarized in Table S4. The most pronounced decline in model performance, particularly in the AUROC of the test and external validation sets, was observed when either basal temperature or the estimated duration of surgery was excluded, highlighting their importance a key predictors. In contrast, excluding any of the other variables resulted in only marginal performance changes, indicating the model demonstrates low dependence on them. This analysis further supports the robustness of the model's predictions and its reliance on the core predictive features.

**Table S4. Comparisons of the model performance with variable exclusion**

| **Characteristics** | **AUROC (95% CI)** | **AUPRC** |
| --- | --- | --- |
| **Exclusion: BMI** |  |  |
| Training set | 0.965 (0.949–0.982) | 0.953 |
| Test set | 0.804 (0.731–0.877) | 0.593 |
| Validation set | 0.886 (0.839–0.932) | 0.811 |
| **Exclusion: ASA physical status** |  |  |
| Training set | 0.975 (0.960-0.990) | 0.966 |
| Test set | 0.795 (0.721-0.869) | 0.585 |
| Validation set | 0.885 (0.837-0.933) | 0.799 |
| **Exclusion:Fasting period** |  |  |
| Training set | 0.974 (0.959-0.989) | 0.964 |
| Test set | 0.791 (0.717-0.866) | 0.578 |
| Validation set | 0.883 (0.834-0.931) | 0.795 |
| **Exclusion: Basal temperature** |  |  |
| Training set | 0.943 (0.920–0.966) | 0.932 |
| Test set | 0.724 (0.626–0.823) | 0.508 |
| Validation set | 0.866 (0.812–0.919) | 0.779 |
| **Exclusion: Estimated duration of surgery** |  |  |
| Training set | 0.944 (0.921–0.967) | 0.925 |
| Test set | 0.779 (0.698–0.861) | 0.577 |
| Validation set | 0.864 (0.811–0.916) | 0.771 |
| **Exclusion: Hypertension** |  |  |
| Training set | 0.975 (0.960–0.990) | 0.966 |
| Test set | 0.794 (0.720–0.868) | 0.579 |
| Validation set | 0.885 (0.838–0.933) | 0.798 |
| **Exclusion: Albumin** |  |  |
| Training set | 0.966 (0.950–0.983) | 0.953 |
| Test set | 0.796 (0.722–0.869) | 0.506 |
| Validation set | 0.885 (0.840–0.934) | 0.792 |
| **Exclusion: ALT** |  |  |
| Training set | 0.973 (0.957–0.989) | 0.963 |
| Test set | 0.778 (0.699–0.857) | 0.569 |
| Validation set | 0.873 (0.821–0.924) | 0.779 |

Abbreviations: BMI=Body mass index, ASA=American society of anesthesiologists, ALT=Alanine aminotransferase, AUROC=area under the receiver operating characteristic curve, AUPRC=area under the precision-recall curve.
